# Light guiding and switching using eccentric core-shell geometries

**DOI:** 10.1038/s41598-017-11401-y

**Published:** 2017-09-11

**Authors:** Ángela I. Barreda, Yael Gutiérrez, Juan M. Sanz, Francisco González, Fernando Moreno

**Affiliations:** 1 Grupo de Óptica, Departamento de Física Aplicada, Universidad de Cantabria, Facultad de Ciencias, Avda. Los Castros s/n, 39005 Santander, Spain; 2 Departamento de I+D, Textil Santanderina, S.A., Avenida Textil Santanderina, s/n, 39500 Cabezón de la Sal, Spain

## Abstract

High Refractive Index (HRI) dielectric nanoparticles have been proposed as an alternative to metallic ones due to their low absorption and magnetodielectric response in the VIS and NIR ranges. For the latter, important scattering directionality effects can be obtained. Also, systems constituted by dimers of HRI dielectric nanoparticles have shown to produce switching effects by playing with the polarization, frequency or intensity of the incident radiation. Here, we show that scattering directionality effects can be achieved with a single eccentric metallo-HRI dielectric core-shell nanoparticle. As an example, the effect of the metallic core displacements for a single Ag-Si core-shell nanoparticle has been analyzed. We report rotation of the main scattering lobe either clockwise or counterclockwise depending on the polarization of the incident radiation leading to new scattering configurations for switching purposes. Also, the efficiency of the scattering directionality can be enhanced. Finally, chains of these scattering units have shown good radiation guiding effects, and for 1D periodic arrays, redirection of diffracted intensity can be observed as a consequence of blazing effects. The proposed scattering units constitute new blocks for building systems for optical communications, solar energy harvesting devices and light guiding at the nanoscale level.

## Introduction

Nanotechnology has revolutionized science of the last decades by generating important theoretical and practical developments. The interaction of electromagnetic radiation with metallic nanoparticles (NPs) has been a vastly investigated field providing applications in many research areas like optics, health, material analysis, communications, biology, etc.^1^. When incident radiation excites a metallic NP, free electrons tend to oscillate at the incident radiation frequency, leading to Localized Surface Plasmons (LSPs). These coherent oscillations of the electronic plasma depend on the optical properties of the NP, its size, shape and the wavelength of the impinging light^2^. For certain frequencies, the energy of the incident light is transferred to free electrons, so that they oscillate with maximum amplitude giving rise to resonances in the scattering spectrum. Although in general, metallic NPs exhibit a good response in the spectral range UV-VIS-NIR, their intrinsic Joule’s losses limit their plasmonic performance in most of applications^3, 4^. High Refractive Index (HRI) dielectric NPs have been proposed instead as ideal candidates for solving this issue^5^ because light can propagate inside them without being absorbed. In addition, they can present magnetic properties in spite of being non-magnetic materials in nature^6^. So, both electric and magnetic resonances can be observed as a consequence of the excitation of whispering gallery modes^7^. Their spectral position and intensity depend on the NP size, its refractive index and that of the surrounding medium, *m*_med_^8, 9^.

When electric and magnetic spectral resonances overlap, coherence effects appear, leading to a real control of the directionality of the scattered light. This has permitted to redirect the incident radiation in forward, backward or at given scattering angles with respect to the incident direction^10–12^. Kerker *et al*.^13^ established that, under certain assumptions of the electric permittivity *ε* and magnetic permeability *μ*, electric and magnetic dipoles can oscillate either in- or out-of-phase. These two situations give rise to special Scattering Directionality Conditions (SDCs), the so-called “zero backward condition” (or First Kerker’s condition) and “minimum forward condition” (or Second Kerker’s condition) respectively^14^.

Recently, symmetric metallo-dielectric or dielectric-dielectric core-shell nanostructures^15–21^ have been studied for improving the scattering directionality properties established by Kerker *et al*.^13^. In particular, by changing the relative size of the core respect to the particle size, it is possible to tune the spectral position of the electric and magnetic resonances and consequently, to govern the above mentioned SDCs^22, 23^. These nanostructures can have applications in solar energy harvesting devices^24–26^, metamaterials^27^ or sensing^22^, among others. More complex geometries, like dimers, trimers or oligomers have also been explored with the same objective^28–30^. Moreover, aggregates of particles have also been proposed to redirect the scattered intensity *I*(*θ*_sca_) into some specific directions respect to that of forward or backward. By means of these structures, it is possible to observe switching effects, whose “on”/“off” states depend on the polarization^31^, frequency^10, 11^ or intensity^32^ of the incident radiation.

Eccentric metallic core-shell nanoparticles (examples are nanoeggs and nanocups configurations) have been analyzed as new plasmonic units providing with both new spectral tuning possibilities and directionality effects. Near and far-field regimes have been studied. Also, the possibility of manufacturing them in a controlled manner, makes these nanounits very attractive for many application purposes where good tunable plasmonic performance is necessary^33–36^. Here, we will pay attention to the electromagnetic behavior of eccentric metallo-dielectric core-shell NPs where the shell is made of a HRI dielectric material.

We show that an isolated eccentric core-shell nanoparticle can operate as an optical switching device in a similar way to the one proposed by Barreda *et al*.^31^, based on an homogeneous dimer made of HRI dielectric NPs. However, the eccentric core-shell configuration presents some advantages respect to the dimer, because only one particle is required. The switching effect will also be analyzed at the 90° scattering configuration, which shows clear advantages with respect to the conventional forward and backward directions^37^.

Another remarkable characteristic of the eccentric core-shell geometry is that, apart from operating as a switching device, it can improve the efficiency of the scattering directionality. In this sense, we demonstrate that there is an optimum core displacement that produces the highest/lowest ratio between the scattered intensities in forward and backward directions for the First/Second Kerker’s condition. By taking advantage of these directionality properties, we have explored the possibility of using aggregates of eccentric core-shell NPs for guiding the incident radiation. A Yagi-Uda type^38^ light guiding scheme has been studied as an example.

The paper is organized as follows: Firstly, we revisit the SDCs for eccentric Ag-Si core-shell NPs for core displacements along the *x*- or *y*-directions, i.e. along the directions perpendicular to that of the propagation, showing a switching effect. Secondly, we analyze the SDCs for core shifts along the propagation direction, improving the efficiency of the scattering directionality patters. Thirdly, we make a brief discussion of the main results and we evidence the possibility of using aggregates of these eccentric core-shell NPs for guiding applications. Finally, we show the methodology used in this research.

## Results

**Figure 1:**
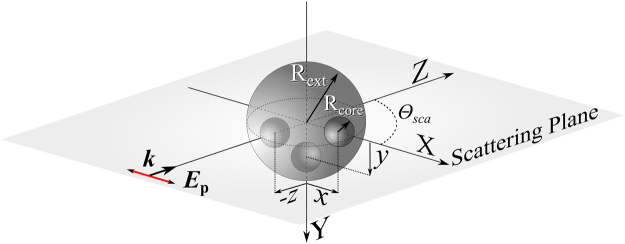
Scattering geometry considered for this study. The core (Ag)-shell (Si) particle has a core radius, *R*_core_, and a shell radius, *R*_ext_. The structure is illuminated with a monochromatic plane wave with wavevector ***k***, along the *z*-axis and polarization, ***E***, along the *x*- or *y*-axis. The three considered displacements are shown by means of the little spheres.

**Figure 2:**
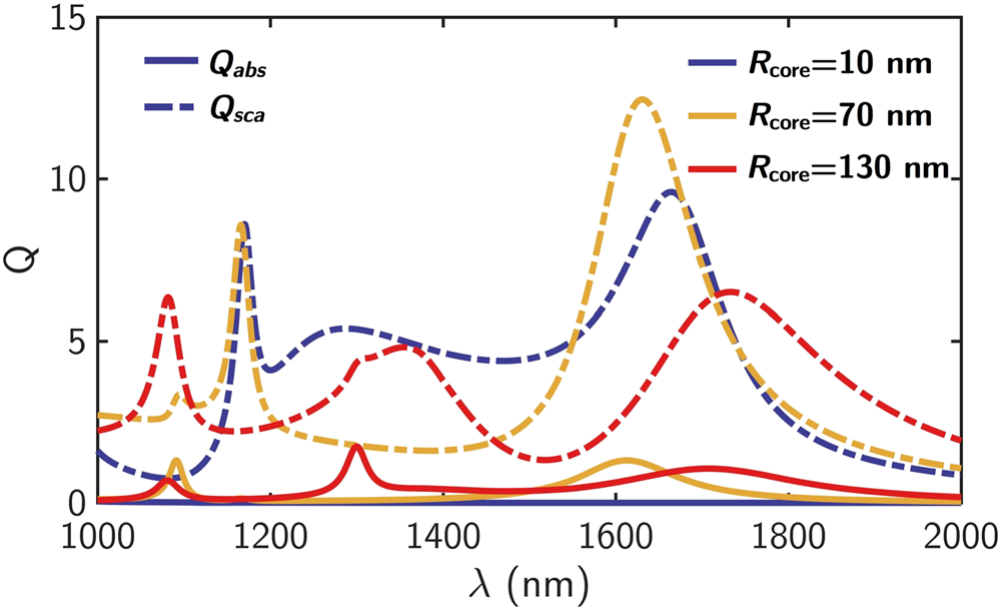
Absorption and scattering efficiencies for a Ag-Si core-shell NP as a function of the core size. Absorption (solid lines) and scattering (dashed lines) efficiencies for various core radii in a Ag-Si core-shell NP (*R*_ext_ = 230 nm), when it is illuminated by a plane wave propagating along the *z*-axis and linearly polarized along the *x*-axis. The values of the analyzed radii are *R*_core_ = 10 nm, 70 nm and 130 nm (blue, yellow and red lines, respectively).

The scattering system is constituted by either isolated or aggregates of eccentric metallo-HRI dielectric core-shell NPs. It is illuminated with a plane wave linearly polarized to either the *x*- or *y*-axis and propagating along the *z*-axis. Its intensity is assumed to be equal to 1. The scattering plane is the *z*-*x* and the displacements of the metallic core inside the NP have been considered to be along the three principal axes, as it is shown in Fig. 1. We have focused on Ag-Si core-shell NPs in which the core has been displaced up to 150 nm along the *x*-, *y*-, *z*-axis from its origin. All the electromagnetic study has been carried out by using Multiple Sphere T-matrix Method (MSTM)^39^ extended to spherical particles with spherical inclusions^40^. We have fixed the external dielectric (Si) shell radius to *R*_ext_ = 230 nm as in previous studies^22^. This size has been chosen because dipolar resonances of pure Si NPs are located in a very specific spectral range (1–2 μm^6^) for radius values between 200–300 nm. This is the spectral range where optical communications devices are commonly designed to operate. Within this region of the spectrum, the imaginary part of the refractive index of pure silicon is negligible, as opposed to what happens in the UV-Visible spectral region^41^. On the contrary, the Ag core is highly absorbing and, although the spectral range is far from Ag plasmonic resonances in vacuum^37, 42^, the high permittivity of the shell allows strong plasma oscillations in the wavelength range studied. In spite of the metallic character of the core, the absorption of the incident radiation in the particles takes low values in the analyzed spectral range. In Fig. 2 we show absorption and scattering efficiencies for a concentric Ag-Si core-shell NP as a function of the metallic core size. It is observed how by increasing the metallic core radius respect to particle size, absorption is only slightly increased, being the scattering the dominant contribution, i.e., Ag core is responsible for absorption in that spectral region. In fact, for *R*_core_ = 10 nm, due to the tiny core size, absorption is negligible as it occurs for pure Si NPs. For eccentric NPs, absorption follows the same behavior, as it can be observed in the Supplementary Note 1, where we show absorption and scattering efficiencies for both different radii of the metallic core and various shifts along the *x*-, *y*- and *z*-axis.

### Revisited Kerker’s conditions for eccentric core-shell NPs

In this section we analyze the SDCs for eccentric metallo-dielectric core-shell NPs as a function of the core shift from the NP center along the *x*-, *y*- or *z*-axis. The study has been performed for different core sizes. In particular, we have considered the following cases: *R*_core_ = 10 nm, 70 nm and 130 nm. Because the electromagnetic response depends on the core displacement direction, we will distinguish two configurations: Shifts of the core either perpendicular or parallel to the propagation direction of the impinging radiation.

#### Core shifts perpendicular to the propagation direction: Switching effects

In this part we revisit the SDCs for an isolated eccentric Ag-Si core-shell NP whose core has been displaced from the NP center along a direction perpendicular to that of the propagation of the incoming radiation, i.e. along *x*-axis in Fig. 1. The SDCs have been numerically obtained because, for this particular geometry, it is not possible to get an analytical solution as for the concentric case. For the smallest core size analyzed (*R*_core_ = 10 nm) both First and Second Kerker’s conditions can be fulfilled. In this case, the scattering diagrams are similar to those corresponding to a concentric core-shell NP regardless of the core displacement. This effect comes from the low influence of the metallic core, as it was pointed out in a previous work^43^. However, for larger core sizes, interesting new effects show up. In fact, when the core displacement is along the *x*-axis, the scattering diagrams rotate with respect to the concentric case. This effect offers the possibility of redirecting the incident radiation at certain angles with respect to either forward or backward directions (First or Second Kerker’s condition, respectively). Also, the rotation is either clockwise or counterclockwise depending on the polarization of the incident radiation and the direction of the shift ( + *x* and -*x* displacements, see Supplementary Note 2). This behavior can be understood looking at the geometrical anisotropy introduced by the core displacement. Core shifts perpendicular to the incident direction provides similar rotation effects to those obtained with a magneto-optical material (a material whose off-diagonal elements in its dielectric tensor are non-negligible^44, 45^). This means that the effective refractive index of the core-shell NP becomes anisotropic due to the symmetry breaking produced by the core shifting.

As the core size increases, electric resonances are red-shifted while magnetic ones are blue-shifted^22^. In particular, focusing on the dipolar spectral region, for *R*_core_ = 10 nm, the magnetic resonance is observed at longer wavelengths than the electric one. Due to the above-mentioned shifts, for *R*_core_ = 70 nm both resonances appear nearly at the same wavelength. Finally, for *R*_core_ = 130 nm, the electric dipolar resonance is red-shifted with respect to the magnetic one. Consequently, only the zero-backward condition (First Kerker’s condition) can be observed for *R*_core_ = 70 nm, whilst only the near zero-forward (Second Kerker’s condition) is attained for *R*_core_ = 130 nm^22, 27^.

**Figure 3:**
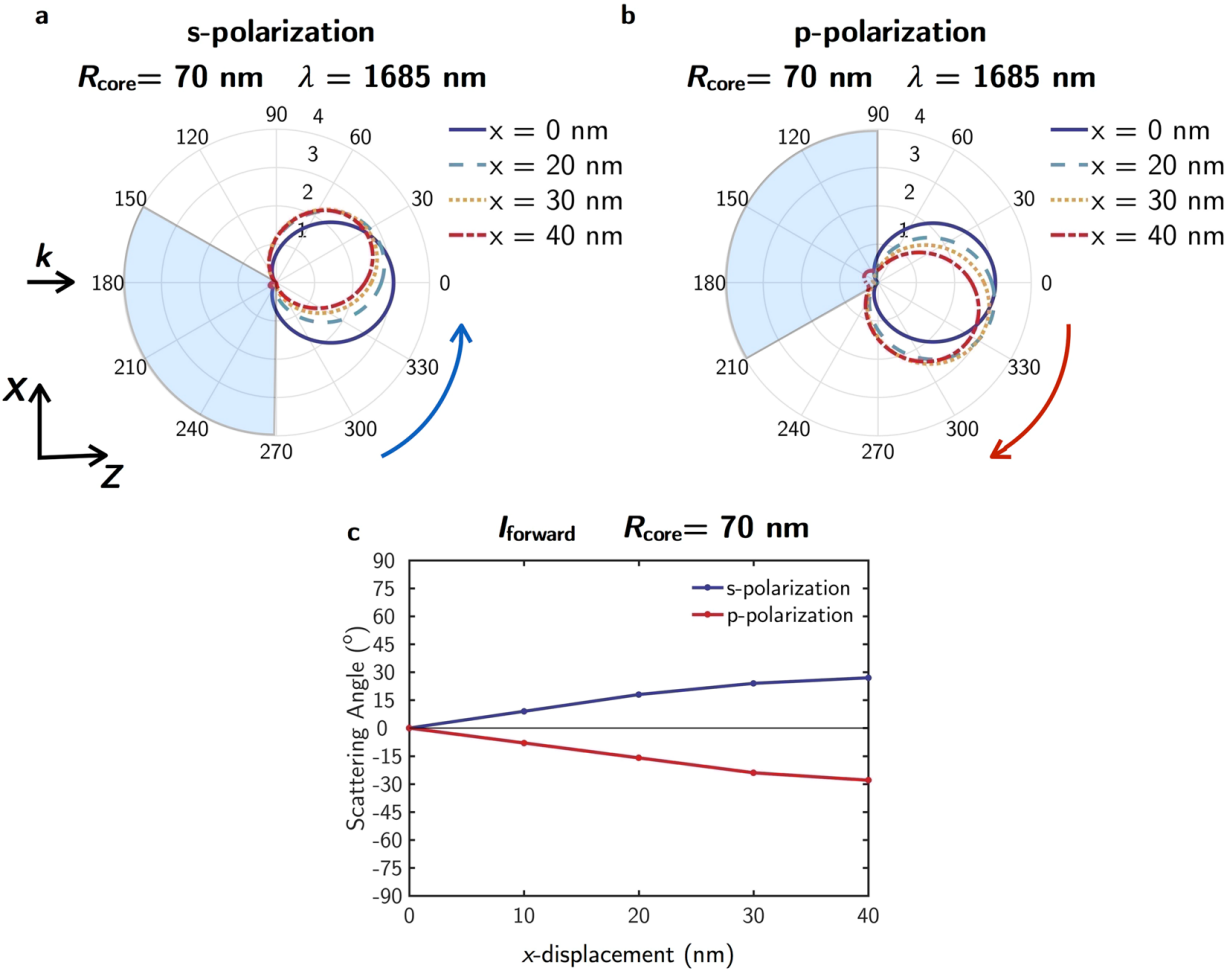
Scattered intensity for *R*_***core***_ = 70 nm as a function of the core displacement. Scattering diagrams for the polarizations of the incident radiation (**a**) perpendicular (s-polarization) and (**b**) parallel (p-polarization) to the scattering plane (*z*-*x* plane in Fig. 1) and different core shifts along the *x*-axis: 0 nm (blue solid line), 20 nm (blue dashed line), 30 nm (yellow dotted line) and 40 nm (red dash-dotted line). The black arrow labelled with ***k*** indicates the propagation direction of the incident beam. The blue and red arrows refer to the rotation direction. (**c**) Angle at which the scattered intensity is maximum as a function of the core displacement along the *x*-axis and s- and p-polarizations of the incident light (blue and red lines respectively).

Figure 3 shows how the maximum scattered intensity at the zero-backward condition (*λ* = 1685 nm) is rotated with respect to the forward direction towards either positive or negative angles depending on the polarization of the incident radiation. When the NP is illuminated by a plane wave linearly polarized along the *y*-axis (s-polarization) the scattering diagram is rotated counterclockwise with respect to the forward direction (see Fig. 3a). On the contrary, when the polarization of the incoming wave is parallel to the core displacement (p-polarization) the rotation is clockwise (see Fig. 3b). The rotation angle depends on the displacement value of the metallic core from the NP center. In Fig. 3c we represent the angle at which the scattered intensity for both s- and p-polarizations is maximum as a function of the core displacement along the *x*-axis. In particular, the largest rotation is for a displacement of 40 nm, obtaining the maximum scattered intensity at approximately 27° or − 28° for s- and p-polarizations respectively. For larger displacements of the core, the SDCs are not longer observed.

As it is shown by means of the shaded gray areas in Fig. 3a,b, it is possible to observe a 120° sector for either s- or p-polarization where the scattered intensity is one order of magnitude lower than in forward direction.

**Figure 4:**
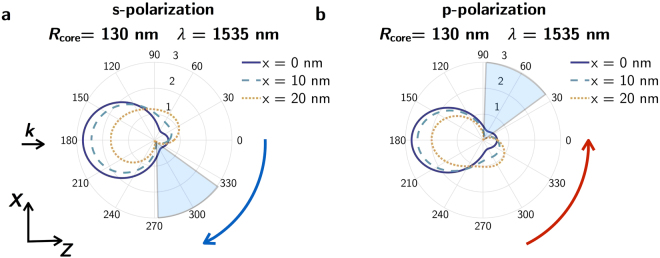
Scattered intensity for *R*_***core***_ = 130 nm as a function of the core displacement. Scattering diagrams for the polarizations of the incident radiation (**a**) perpendicular (s-polarization) and (**b**) parallel (p-polarization) to the scattering plane (*z*-*x* plane in Fig. 1) for different core shifts along the *x*-axis: 0 nm (blue solid line), 10 nm (blue dashed line) and 20 nm (yellow dotted line). The black arrow labelled with ***k*** indicates the propagation direction of the incident beam. The blue and red arrows refers to the rotation direction.

Figure 4 shows the scattering diagrams for the s- (a) and p-polarization (b) of the incident radiation, and different displacements along the *x*-axis for *R*_core_ = 130 nm. As stated before, for this core size, only the near zero-forward condition (*λ* = 1535 nm) is achieved. In fact, for *x* = 20 nm, even this condition tends to disappear. Similarly to the case of the zero-backward condition for *R*_core_ = 70 nm, for *R*_core_ = 130 nm, it is possible to observe a rotation of the scattering diagrams at the wavelength where the near zero-forward condition appears, which is clockwise or counterclockwise for s- and p-incident polarization, respectively. Due to this rotation, there is a 50° sector where the scattered intensity is one order of magnitude lower than in backward direction. This region (shaded in grey) is attained for negative or positive angles depending on the polarization of the incident radiation.

This rotation effect makes these structures very promising for switching purposes. By only changing the polarization of the incident radiation, it is possible to go from “on” to “off” states. In particular, we show that the scattered intensity at 90° with respect to the incident direction can be tuned from almost null to maximum values by playing with the polarization of a single frequency excitation and with only an eccentric metallo-dielectric core-shell NP.

**Figure 5:**
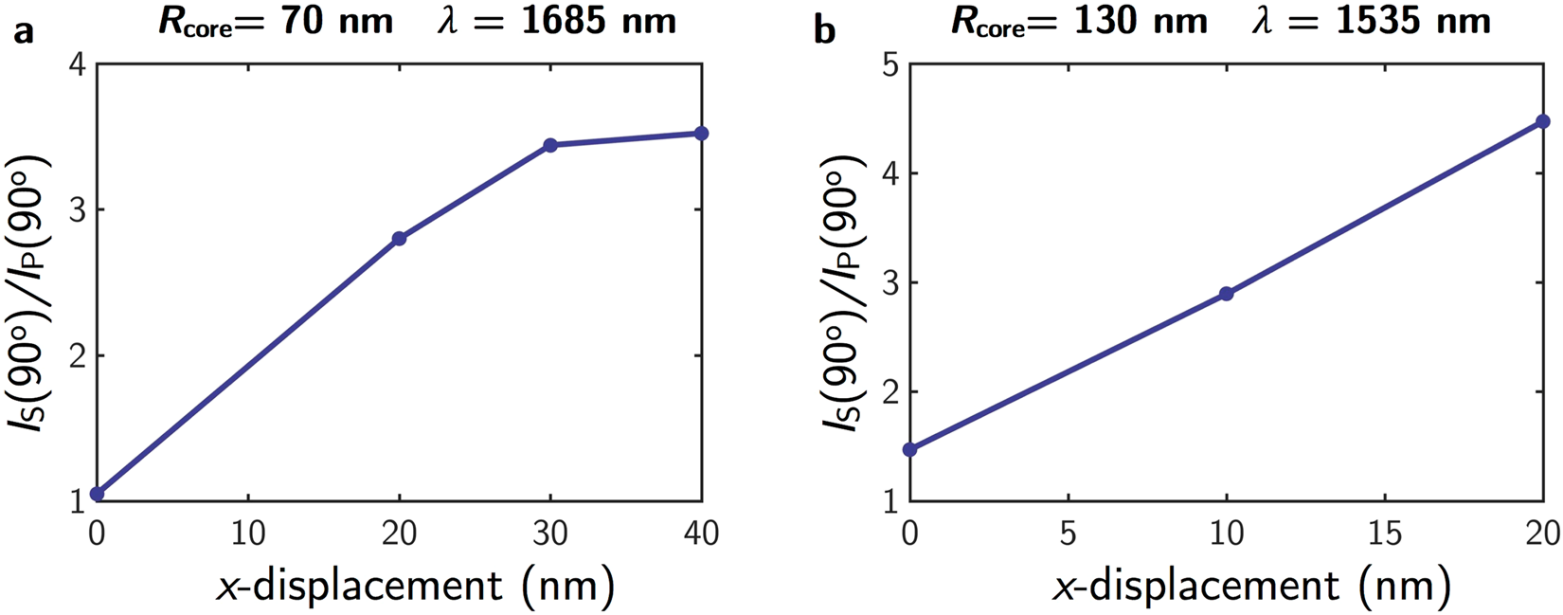
Ratio I_**S**_/I_**P**_ at 90° scattering angle. *I*_S_(90°)/*I*_P_(90°) as a function of the core displacement along the *x*-axis for different core sizes for the wavelengths corresponding to the two SCDs. (**a**) *R*_core_ = 70 (First Kerker’s condition) nm and (**b**) *R*_core_ = 130 nm (Second Kerker’s condition) respectively.

In order to show more clearly the above mentioned switching effect, in Fig. 5 we plot *I*_S_(90°)/*I*_P_(90°), the ratio between the scattered intensity at 90° for s- and p-incident polarizations and the wavelengths corresponding to the two SDCs. For both core sizes, 70 nm and 130 nm, as the displacement of the core along the *x*-axis increases, that ratio takes larger values. In particular, for *R*_core_ = 70 nm (Fig. 5a) we obtain a ratio of 3.5 for a displacement of *x* = 40 nm. For *R*_core_ = 130 nm, the ratio for the largest displacement, *x* = 20 nm, is 4.5. These results demonstrate that by swapping the polarization of the exciting radiation from s to p, and fixing a minimum intensity threshold (which will be considered as the “off” state of the device), the scattered radiation at 90° can go from “on” to “off” states. Detecting at 90° is a good way to avoid any parasitic effect due to the incident radiation. In addition, incident polarization is conserved in the scattered radiation when the core shifts are in the scattering plane. Note that results for displacements along the *x*/*y*-axis and p/s-polarizations are equivalent to those corresponding to *y*/*x*-axis and s/p-polarizations.

#### Core shifts parallel to the propagation direction: Improving the SDCs

**Figure 6:**
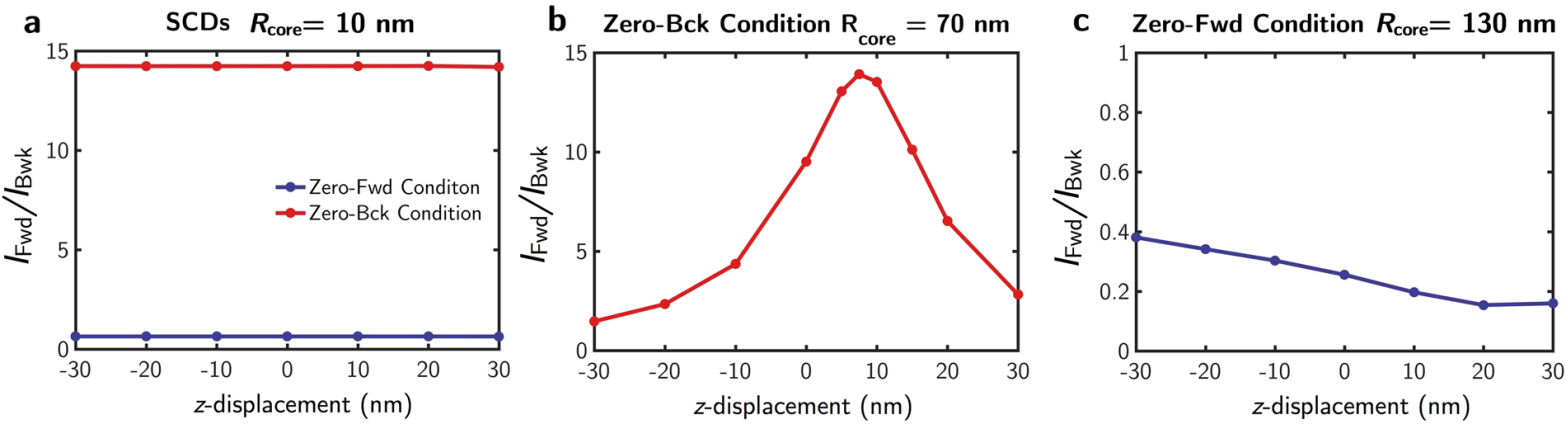
Integrated intensity ratios. Ratio between the integrated scattered intensity in the forward (*θ*_sca_ ∈[−90°, 90°]) and backward angular regions (*θ*_sca_∈[90°, 270°]) as a function of the core displacement along the *z*-axis for different core sizes on the First/Second Kerker’s condition. (**a**) *R*_core_ = 10 nm, (**b**) *R*_core_ = 70 nm and (**c**) *R*_core_ = 130 nm.

In this section we analyze the electromagnetic behavior of eccentric core-shell NPs when the core displacement is along the *z*-axis (along the propagation direction). In this case, the rotation is not longer achieved because there are no breaks in the symmetry of the problem with respect to either the *x*- or *y*-axis, i.e. with respect to p- or s-polarization directions (results are identical for both polarizations of the incident radiation, p and s). However, directionality effects can still be observed. In order to enhance these effects on the analyzed nanostructures, we have looked for the core displacement that produces the highest/lowest ratio between the integrated scattered intensity in the forward and backward angular regions at the First/Second Kerker’s condition for all the analyzed core sizes: *R*_core_ = 10 nm (*λ* = 1535 nm, Second Kerker’s condition) and (*λ* = 1820 nm, First Kerker’s condition), *R*_core_ = 70 nm (*λ* = 1685 nm, First Kerker’s condition) and *R*_core_ = 130 nm (*λ* = 1535 nm, Second Kerker’s condition). See Fig. 6a–c respectively. For *R*_core_ = 10 nm the ratio is independent of the core displacement. This is because of the small volume of the core in comparison to the volume of the whole particle. Therefore,its influence in the SDCs is negligible^43^. Results are similar to those corresponding to an isolated particle of radius *R* = 230 nm made of pure silicon. However, for *R*_core_ = 70 nm, the largest ratio is obtained for *z* = +7.5 nm. For this *z*-displacement, the integrated scattered intensity in the forward angular region is 14.0 times larger than that scattered in the backward region. This enhances the First Kerker’s condition with respect to a concentric core-shell case (*z* = 0 nm). Furthermore, the integrated scattered intensity ratio between forward and backward angular regions is similar to that obtained for the isolated particle (in fact, this value is 14.2). This is shown in Fig. 6a. This suggests that for a particular core size (*R*_core_ = 70 nm in this case) and a given shift of the metallic core (*z* = +7.5 nm in this case), it is possible to obtain the same results as for a pure Si nanoparticle, where losses are negligible in the analyzed spectral range. This is to say that losses introduced by the metallic core are minimized. For *R*_core_ = 130 nm, the lowest ratio is observed for *z* = +20 nm. In this case, the intensity scattered in the forward angular region is 0.15 times smaller than that scattered in the backward direction. This enhances the Second Kerker’s condition with respect to the concentric core-shell case.

## Discussion

The influence of shifting the metallic core in a metal-HRI dielectric (Ag-Si for the analyzed case) core-shell NP has been deeply analyzed. For practical purposes linked to telecommunications applications, the external radius has been fixed to *R*_ext_ = 230 nm while the core size (*R*_core_) has been varied from 10 nm to 130 nm. We have studied the scattering properties of these scattering units by looking at the angular distribution of the scattered intensity in the far-field. For the smallest core size (10 nm) it is not observed any significant change due to the smallness of the core in comparison to the size of the whole particle, 460 nm. However, as the core size increases, some interesting properties arise for the different core displacements. For core displacements perpendicular to the propagation direction of the incoming radiation, a rotation of the SDCs is observed. Its direction depends on the polarization of the incident radiation. In particular, for *R*_core_ = 70 nm, the rotation of the scattering diagrams at the First Kerker’s condition is clockwise for p-incident polarization and counterclockwise for s-incident polarization. The opposite behaviour is found for *R*_core_ = 130 nm, being the rotation at the Second Kerker’s condition counterclockwise for p-incident polarization and clockwise for s-incident polarization. This rotation effect, which is only manifested for the largest core sizes, makes eccentric metallo-dielectric core-shell NPs interesting scattering units for redirecting the incident radiation in the desired angular range and also for building optical switching devices. The switching effect has been quantified by the ratio of the scattered intensity at 90° for the analyzed incident polarizations, either s or p. The large values obtained for *I*_S_(90°)/*I*_P_(90°) demonstrate that by swapping the polarization of the impinging radiation from s to p, and fixing a minimum intensity threshold (“off” state), the scattered radiation at 90° could go from “on” to “off” states.

The fact that only the First or Second Kerker’s conditions can be obtained for *R*_core_ = 70 nm or *R*_core_ = 130 nm is due to the red-shift and blue-shift undergone by the electric and magnetic resonances as the core size increases, respectively.

Concerning core displacements parallel to the propagation direction, for certain *z*-axis shifts, the efficiency of the SDCs can be enhanced. In fact, the ratio between the integrated scattered intensity in the forward and backward angular regions can reach the highest/lowest value for *z* = 7.5 nm/*z* = 20 nm and *R*_core_ = 70 nm/*R*_core_ = 130 nm for the wavelength where the First/Second Kerker’s condition is observed.

In order to enhance the analyzed effects, aggregates of the studied eccentric NPs can be used to build nanoantennas for switching and radiation guiding at the nanoscale. To explore this possibility, we have envisaged two types of configurations: chains of up to 6 particles in different spatial distributions and 1D periodic arrays. For the former, this number of particles has been chosen to maintain reasonable directionality and switching conditions as well as to enhance the scattered intensity (see Supplementary Note 3 for further details). Each core-shell NP in the chain has an external and core radius of *R*_ext_ = 230 nm and *R*_core_ = 70 nm, respectively. The core has been displaced 30 nm along the *x*-direction.

**Figure 7:**
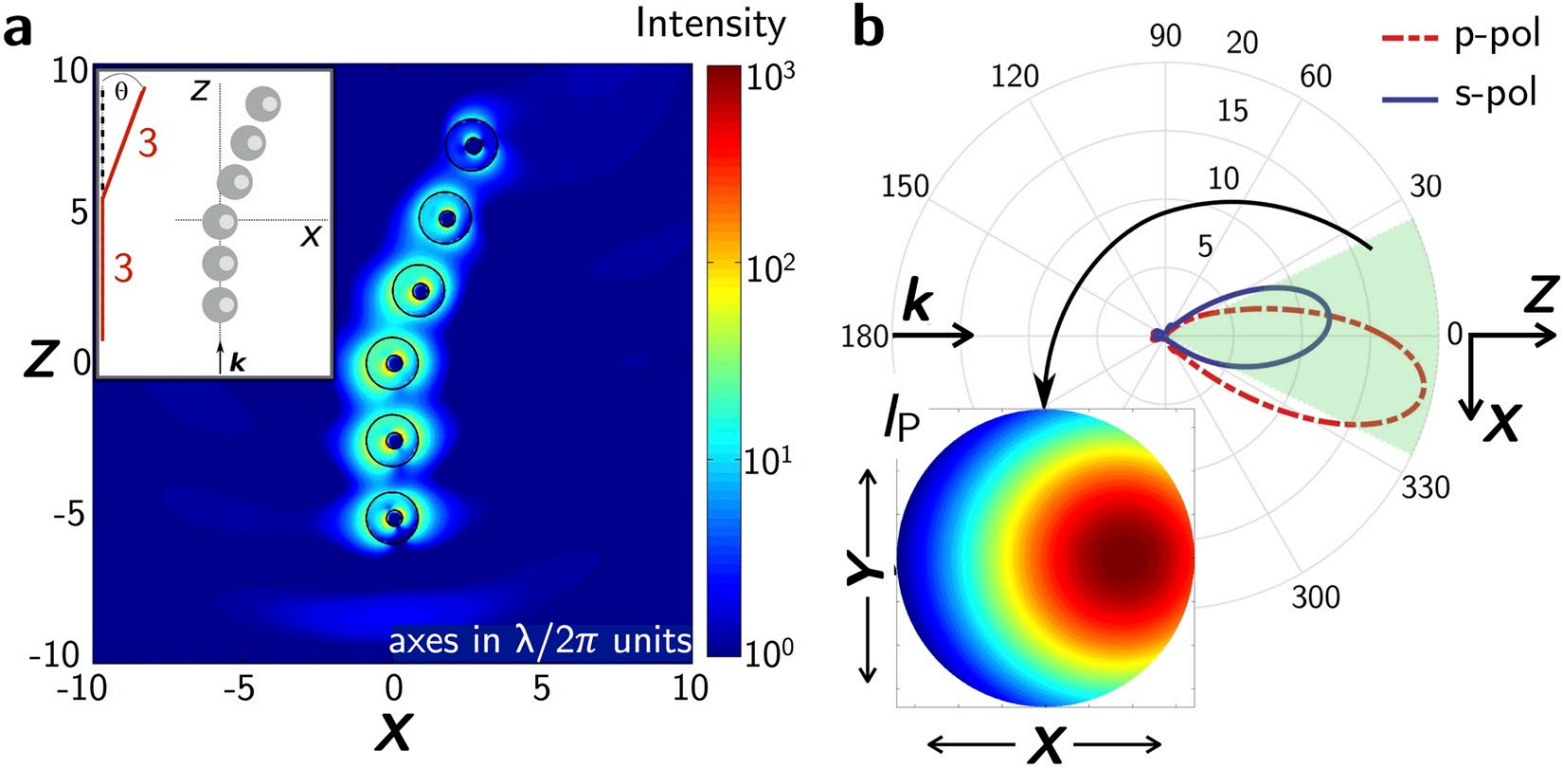
Near- and far-field behaviour of a chain of particles. (**a**) shows near field map of a V-shaped chain of 6 core-shell (Ag-Si) nanoparticles of radii *R*_ext_ = 230 nm and *R*_core_ = 70 nm with the core displaced 30 nm along the *x*-axis. Inset is a scheme of the geometry. The distance between the particles along the *x*-axis is 137 nm. The angle between segments of aligned NPs is *θ* = 20° (see inset in (**a**)). The structure is illuminated with a plane wave propagating along the *z*-axis and polarized along the *x*-axis (p-polarization). The plot corresponds to the *z*-*x* plane for the wavelength where the Zero-Backward condition holds for the isolated particle. (**b**) Scattering diagrams in the *z*-*x* plane for p-incident polarization (red dashed-dotted line) and s-incident polarization (blue solid line) at the First Kerker’s condition for the geometry shown in the inset of (**a**). Inset shows far-field observation of the normalized total scattered intensity, within a solid angle of 30°, for p-polarized incident radiation.

In Fig. 7a, the near field map corresponding to a V-shaped chain of 6 particles at the wavelength where the Zero-Backward condition (*λ* = 1685 nm) holds for the isolated particle is shown. The inset shows the spatial configuration of the particles. The angle between segments of aligned NPs has been chosen to be *θ* = 20° in order to take advantage of the rotation observed in the scattering diagrams of an isolated core-shell NP (see Fig. 3c). The distance between the particles is 137 nm in analogy with a previous work^46^. Figure 7b plots the angular distribution of the far-field scattered intensity for both p- and s-incident polarizations at *λ* = 1685 nm (wavelength where the First Kerker’s condition holds for the isolated particle). The inset of Fig. 7b plots the far-field observation of the total scattered intensity, within a solid angle of 30°, for p-polarized incident radiation.

In Fig. 7, it can be seen how this chain of particles is able to interact with the impinging radiation maintaining a good intensity enhancement and narrow scattering patterns. Obviously, these structures are more complex than isolated eccentric NPs, but this is balanced with the scattered intensity gain (mainly in p-/s-polarization for *z*-*x*/*z*-*y*-plane geometries respectively, see Supplementary Note 3) and the strong and narrow scattering patterns. Regarding the values of the scattered intensity, they are higher for the antenna than for the isolated core-shell due to the larger number of NPs that contribute to the scattered radiation. The fact that incident radiation can be guided into the desired direction through aggregates of eccentric core-shell NPs evidences the great potential directionality properties of these nanostructures, opening a door to new antenna designs.

**Figure 8:**
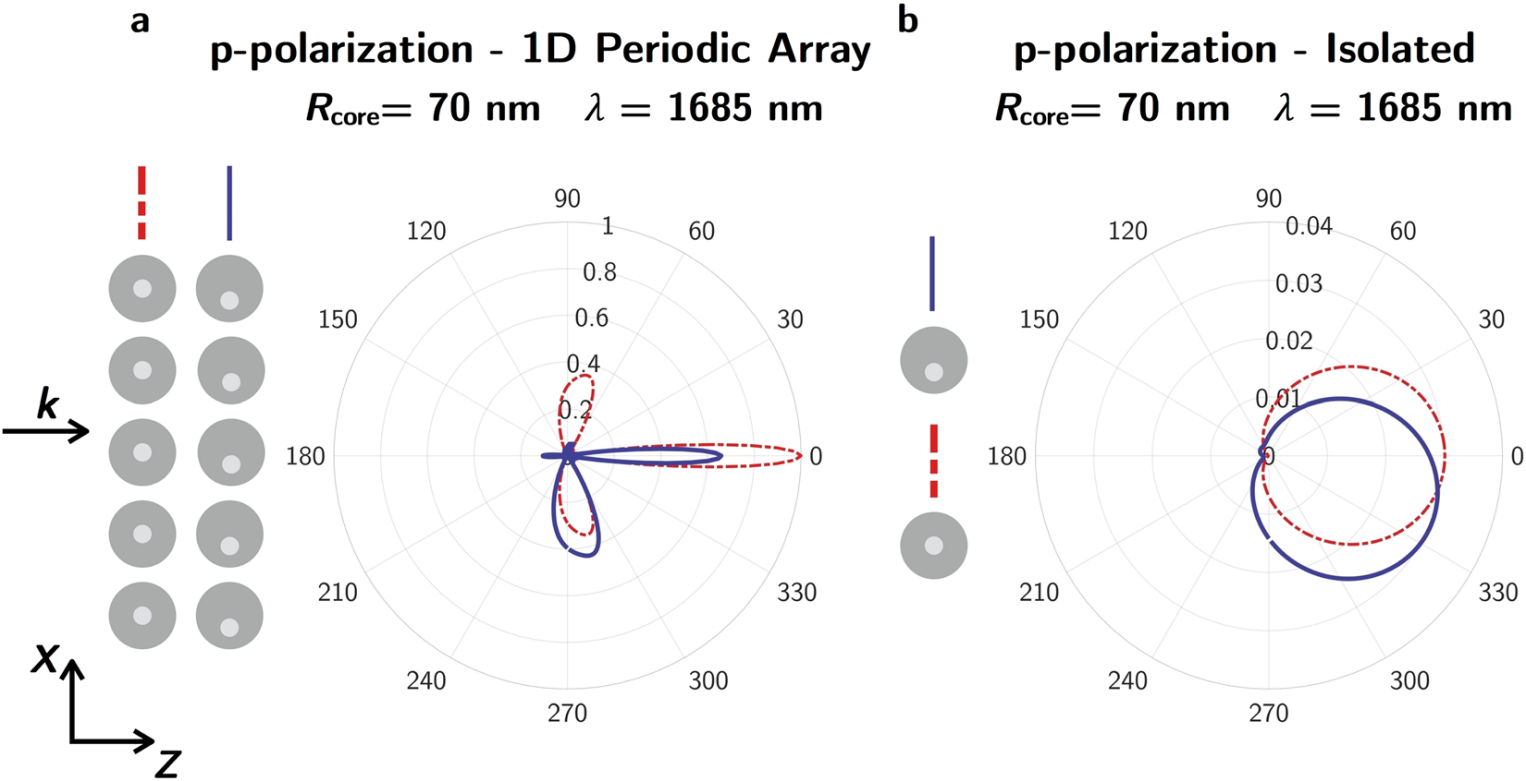
Far-field scattering by a 1D periodic array of eccentric core-shell (Ag-Si) NPs. Scattering diagrams for the polarization parallel to the scattering plane (p-polarization) for: (**a**) five concentric (red dashed-dotted line) and eccentric (solid blue line) core-shell (Ag-Si) NPs in a 1D periodic array configuration with a period of 1711 nm and (**b**) an isolated concentric (red dashed-dotted line) and eccentric (solid blue line) core-shell (Ag-Si) NP. In both cases, *R*_core_ = 70 nm. For the eccentric case, the core has been shifted 30 nm along the polarization direction (*x*-axis). The numerical data have been normalized to the maximum intensity scattered by the 1D periodic array structure built with concentric spheres.

Figure 8a shows the far-field scattering patterns of a system built with 5 core-shell NPs (Ag-Si, *R*_ext_ = 230 nm and *R*_core_ = 70 nm), either concentric or eccenctric, distributed as a 1D array with a period of 1711 nm. The system is illuminated by a plane wave linearly polarized along the direction that connects the NPs, p-incident polarization. For the eccentric core-shells, the core has been shifted 30 nm along the polarization direction. This 1D periodic array configuration tries to mimic a periodic diffraction grating. For comparison, it is also shown the scattering patterns of the corresponding isolated particles, Fig. 8b. Using this 1D periodic array of eccentric NPs, it is possible to obtain a “blazing” effect and, consequently, a redirectionality of the diffracted radiation due to the scattering anisotropy of the isolated particle. A narrowing of the scattering pattern lobes is expected as the number of array elements is increased.

Finally, it is important to point out that the synthesis of isolated eccentric core-shells^47, 48^ and the fabrication of periodic arrays made of either homogeneous^49^ or inhomogeneous^33, 34^ (including both concentric and eccentric core-shells) has already been achieved. However, the construction of these antennas remains a challenge since it needs a precise alignment of the cores and particles.

## Methods

### Lorenz-Mie theory

According to the Lorenz-Mie theory^50^, the incident field can be expanded into vector spherical harmonics. Two cases can be considered, one in which the electric field is polarized perpendicular to the scattering plane (*E*_s,inc_) and one in which the electric field lies within the scattering plane, (*E*_p,inc_). The scattered electric-field components are related to the incident ones via the scattering amplitude matrix:

1 $$(\begin{array}{c}{E}_{p,\mathrm{sca}}\\ {E}_{s,\mathrm{sca}}\end{array})\propto (\begin{array}{cc}{S}_{{\rm{2}}} & {S}_{{\rm{3}}}\\ {S}_{{\rm{4}}} & {S}_{{\rm{1}}}\end{array})(\begin{array}{c}{E}_{p,\mathrm{inc}}\\ {E}_{s,\mathrm{inc}}\end{array})$$

In case of homogeneous isotropic non-active spheres *S*_1_, *S*_2_, *S*_3_ and *S*_4_ are given by

2 $$\{\begin{array}{c}{S}_{{\rm{1}}}=\sum _{n}\frac{2n+1}{n(n+\mathrm{1)}}({a}_{{\rm{n}}}{\pi }_{{\rm{n}}}+{b}_{{\rm{n}}}{\tau }_{{\rm{n}}}),\\ {S}_{{\rm{2}}}=\sum _{n}\frac{2n+1}{n(n+\mathrm{1)}}({a}_{{\rm{n}}}{\tau }_{{\rm{n}}}+{b}_{{\rm{n}}}{\pi }_{{\rm{n}}}),\\ {S}_{{\rm{3}}}={S}_{{\rm{4}}}\mathrm{=0}\end{array}$$

where *π*_n_ and *τ*_n_ are the angular functions, and *a*_n_, *b*_n_ are the so-called Lorenz-Mie scattering coefficients, i.e. the weights of electric and magnetic modes, respectively, that appear in the linear expansion of vector spherical harmonics and indicate the strength of the multipolar contributions of order *n*. These coefficients depend on the electric and magnetic properties of the particle, on the surrounding medium and on the size parameter *x* = 2*πRm*_med_/*λ*, where *λ* is the wavelength of the incident light in vacuum and *R* is the radius of the spherical particle. In particular, *a*_1_ and *b*_1_ correspond to the electric and magnetic dipolar modes and *a*_2_ and *b*_2_ to the quadrupolar ones, respectively.

The total scattered intensities with polarization parallel, *I*_P_(*θ*_sca_), and perpendicular, *I*_S_(*θ*_sca_), to the scattering plane are proportional to |*S*_2_(*θ*)|^2^ and |*S*_1_(*θ*)|^2^, respectively, whereas extinction and scattering efficiencies are given by:

3 $$\begin{array}{c}{Q}_{{\rm{ext}}}=\frac{2}{{x}^{2}}\sum _{n\mathrm{=1}}^{\infty }\mathrm{(2}n+\mathrm{1)}Re\{{a}_{{\rm{n}}}+{b}_{{\rm{n}}}\}\\ {Q}_{{\rm{sca}}}=\frac{2}{{x}^{2}}\sum _{n\mathrm{=1}}^{\infty }\mathrm{(2}n+\mathrm{1)}(|{a}_{{\rm{n}}}{|}^{2}+|{b}_{{\rm{n}}}{|}^{2})\end{array},$$

Nevertheless, eccentric core-shell NPs are a particular case of spherical NPs and cannot be analyzed as homogeneous non-active isotropic spheres^51–53^, in such a way that *S*_3_ and *S*_4_ can be different from 0, depending on the scattering direction and system geometry. A deeper theoretical analysis to obtain the scattering amplitude matrix elements in such systems is avoided, since MSTM gives conspicuous results. Further details about scattering theory of anisotropic spheres can be found in the bibliography^54, 55^.

### MSTM and FEM

The scattering patterns at different wavelengths and for both analyzed polarizations of the incident radiation (s and p) have been studied by using the T-matrix method^39^. T-matrix is one of the most powerful and widely used tools for accurately computing light scattering by particles, both isolated and aggregated, based on directly solving Maxwell’s equations. In this method, the field external to the cluster of *N*-spheres (***E***_sca_) is represented by the superposition of the incident (***E***_inc_) and scattered fields, and the scattered field consists of components radiated from each sphere in the target (***E***_sca,i_):

4 $${{\bf{E}}}_{{\rm{sca}}}={{\bf{E}}}_{{\rm{inc}}}+\sum _{i=1}^{{N}_{{\rm{tot}}}}{{\bf{E}}}_{\mathrm{sca},i}$$

Incident and scattered fields can be represented by regular and outgoing vector spherical wavefunction expansions, leading to an extension of Lorenz-Mie theory to the multiple sphere system. Application of the proper continuity equations at the surface of each sphere results in a system of interaction equations for the scattered field coefficients, where the boundaries are in the form of closed spherical surfaces^56^. Although the code allows for Gaussian Beam incidence, all calculations have been done using plane waves, to avoid singularities and misunderstandings related to scattering patterns.

In the case of the 1D periodic arrays, the electromagnetic simulations have been made by means of the Finite Element Method (FEM) implemented on the commercial software COMSOL Multiphysics^57^. In particular, we used the RF Module that allows us to formulate and solve the differential form of Maxwell’s equations together with the initial and boundary conditions. The equations are solved using the finite element method with numerically stable edge element discretization in combination with state-of-the-art algorithms for preconditioning and solution. A spherical region of embedding medium around the NP is also modeled, whose radius is larger than a wavelength. A perfectly matched layer (PML) domain is outside of the embedding medium domain and acts as an absorber of the scattered field. The mesh was fine enough as to allow convergence of the results.

## Electronic supplementary material

Supplementary Information
