# Supplementary material for: Light guiding and switching using eccentric core-shell geometries
Source: Sci Rep. 2017 Sep 11;7:11189. doi: 10.1038/s41598-017-11401-y (PMC5593893; doi:10.1038/s41598-017-11401-y)
Supplement: Supplementary file 1 — Supplementary Information [file 41598_2017_11401_MOESM1_ESM.doc]

**Light guiding and switching using eccentric core-shell geometries**

Ángela I. Barreda^1^, Yael Gutiérrez^1^, Juan M. Sanz^1,2^, Francisco González^1^, and Fernando Moreno^1,*^

^1^Grupo de Óptica, Departamento de Física Aplicada, Facultad de Ciencias, Universidad de Cantabria, Avda. Los Castros s/n, 39005, Spain

^2^Departamento de I+D, Textil Santanderina, S.A., Avenida Textil Santanderina, 39500, Cabezón de la Sal, Spain

^*^Corresponding author: morenof@unican.es

**Supplementary Note 1**

**Absorption and scattering efficiencies: influence of the core size, polarization of the incident radiation and core displacement:** We have analyzed a core size range from *R*_core_ = 10 nm to *R*_core_ = 130 nm. For the smallest cores, their presence is not significant in the resulting spectra. For that reason, moving the core along the three main axes of the NP provides almost the same spectral behavior of the efficiencies as those of the concentric case. Furthermore, the total amount of metal in the particle is far from being 1%, so the influence of the metal in the particle absorption is negligible^1^.

**Supplementary Figure S1 | Absorption and scattering efficiencies: *x*- and *y*-displacements.** Absorption (solid lines) and scattering (dashed lines) efficiencies for various *x*- and *y*-axis shifts of the core in a Ag-Si core-shell NP (*R*_ext_ = 230 nm), when it is illuminated by a plane wave propagating along the *z*-axis and linearly polarized along the *x*-axis (p-polarization) or *y*-axis (s-polarization). Core radius is *R*_core_ = 70 nm for the left plots **(a, c)** and *R*_core_ = 130 nm for the right ones **(b, d)**. *x*-displacements under p-polarization are equivalent to *y*-displacements under s-polarization. Also, *x*-displacements under s-polarization are equivalent to *y*-displacements under p-polarization.

As the core grows, the electromagnetic behavior becomes more complex (Fig. S1). For the particular case of *R*_core_ = 70 nm, in the dipolar region (λ > 1500 nm), we only observe one peak due to the coupling of the electric and magnetic dipolar resonances because of their spectral shifting. The main effect of the core displacements can be observed in the scattering efficiencies. These take lower values at the frequencies where the dipolar resonances appear as the core displacement increases. Besides this, the resonances get broader. The described consequences are more notorious for core shifts along the direction of polarization. Regarding the absorption efficiency and for the largest displacements, it takes lower values for the polarization perpendicular to the core movement. For bigger radii, the combined dipolar peak splits totally, and the coupling between the electric and magnetic dipolar resonances disappears. In fact, for *R*_core_ = 130 nm we observe two different resonances, one on the right that corresponds to a dipolar electric resonance, and another on the left that is mainly due to the dipolar magnetic contribution. This behavior is different to that observed for *R*_core_ = 10 nm, where the dipolar magnetic resonance is redshifted with respect to the electric one. The differences are obviously caused by the spectral red- and blue-shifts of the electric and magnetic resonances respectively. Focusing on the dipolar region, it is possible to conclude that the scattering resonances take lower values for *R*_core_ = 130 nm than for *R*_core_ = 70 nm because of the decoupling between the electric and magnetic resonances.

Because the incident light propagates along the *z*-axis, results observed for positive and negative movements of the core along the *x*- or *y*-axis with respect to the concentric core-shell NP, show similar behavior. However, when the core is moved along the *z*-direction, positive and negative displacements give different results, see Fig. S2. But, in this case, the direction of the polarization does not influence the results, as it is expected attending to symmetry reasons. For *R*_core_ = 10 nm, due to the low influence of the small metallic core, the results obtained are identical, independently of the core shift. For *R*_core_ = 70 nm and a core shift corresponding to *z* = ± 30 nm, the electric and magnetic dipolar resonances uncouple, and two different resonances are observed in the spectra. In the case of *z* = +30 nm, the absorption is lower than for *z* = -30 nm.

**Supplementary Figure S2| Absorption and scattering efficiencies: +/- *z*-displacements**. Absorption (solid lines) and scattering (dashed lines) efficiencies for various *z*-axis shifts of the core in a Ag-Si core-shell NP (*R*_ext_ = 230 nm), when it is illuminated by a p-polarized plane wave. *R*_core_ = 10 nm, 70 nm and 130 nm for **a** to **c**, respectively.

**Supplementary Note 2**

**Analysis of the rotation of the scattering diagrams as a function of the polarization of the illuminating beam and the direction of the core displacement:** We have analyzed how the scattering diagrams for eccentric metallo-dielectric core-shell nanoparticles with the core shifted along the *x*-direction are rotated either clockwise or counterclockwise depending on the polarization of the illuminating beam. Although shifting the core in the *x*-axis (+*x* or –*x* displacements) does not have any effect on the absorption and scattering cross-sections, it also produces a rotation of the scattering diagrams. Figure S3 shows how the rotation direction of the scattering diagrams (*λ* = 1685 nm) of a core-shell with *R*_core_ = 70 nm can be switched from clockwise to counterclockwise (or vice versa) by changing the polarization of the illuminating beam (from p- to s-polarization for +*x* displacements or from s- to p-polarization for –*x* displacements) or the direction of the core shift (from +*x* to –*x* displacements for p-polarization or from –*x* to +*x* for s-polarization).

**Supplementary Figure S3 | Scattered intensity diagrams for *R*_core_ = 70 nm as function of the displacement direction and polarization of the illuminating beam.** Scattering diagrams for the polarizations of the incident radiation (**a, b**) parallel (p-polarization) and perpendicular (**c, d**) (s-polarization) to the scattering plane (*z-x* plane) and for different core displacements along (**a, c**), and in the opposite direction (**b, d**) to the *x*-axis. The black arrow labelled with ***k*** indicates the propagation direction of the incident beam.

Figure S4 shows how the near-field intensity enhancement map is also rotated when the core is displaced along the *x*-direction. This rotation switches its direction when the displacement is along (Fig. S4(a)) or opposite to (Fig. S4(b)) the *x*-axis. Although we only show the near fields corresponding to p-polarization, this rotation can be also observed for s-polarization. However, the direction of the rotation for s-polarization and positive/negative displacements is equivalent to negative/positive shifts for p-polarization.

**
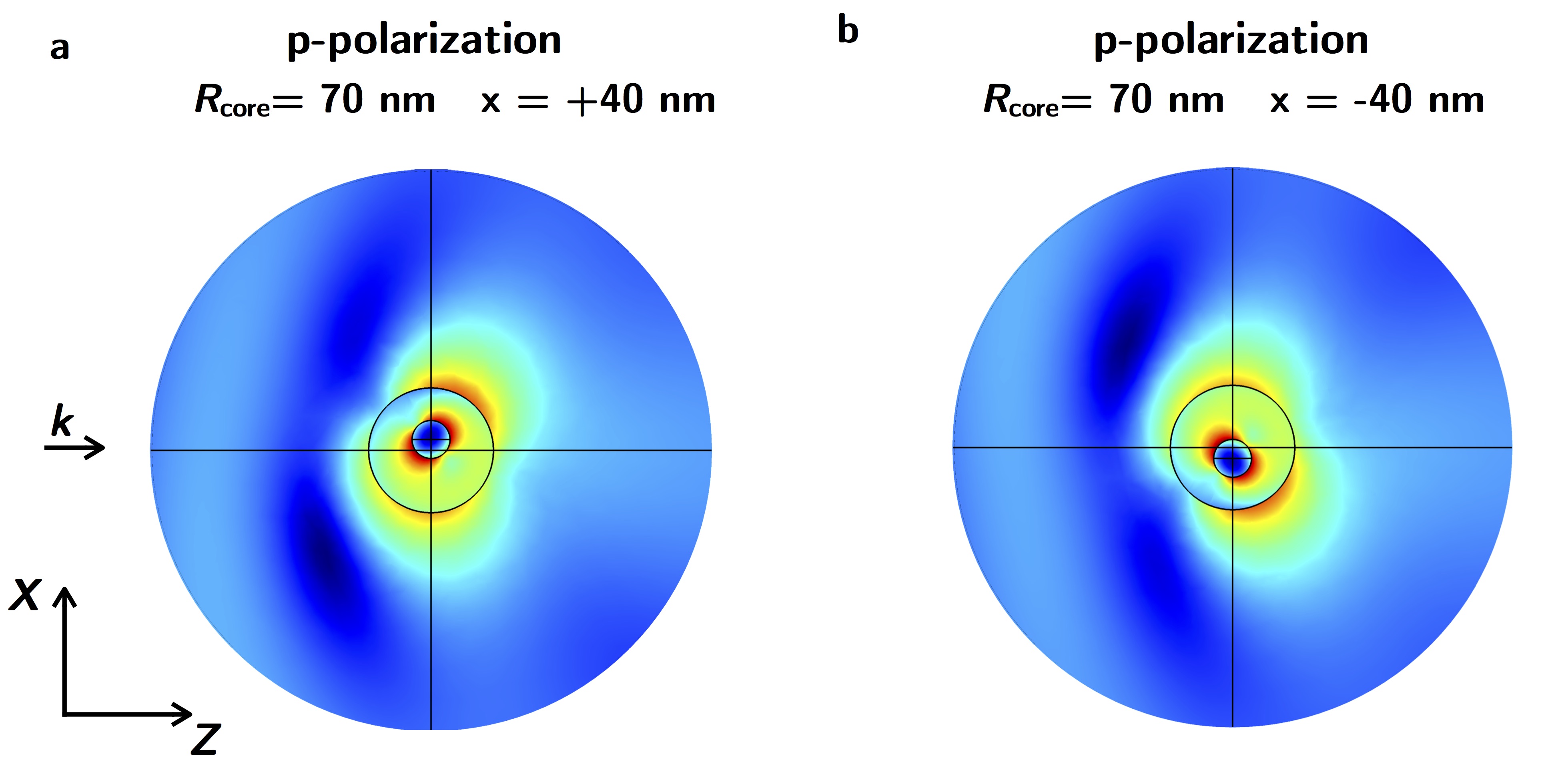
**

**Supplementary Figure S4 | Near-fied intensity maps for *R*_core_ = 70 nm as function of the displacement direction.** Near-field intensity distribution (logarithmic scale) for isolated eccentric core-shell nanoparticles whose core has been shifted along (**a**) and opposite direction (**b**) to the *x*-axis direction. The black arrow labelled with ***k*** indicates the propagation direction of the incident beam and the illuminating wavelength is *λ*=1685 nm.

**Supplementary Note 3**

**Analysis of scattered intensity, *I*(*θ*_sca_), by eccentric core-shell aggregates:** Scattering diagrams corresponding to configurations of up to 6 eccentric core-shell nanoparticles (see Fig. S6) for polarizations of the incident radiation parallel (p-polarization) and perpendicular (s-polarization) to the scattering plane at the wavelength where the Zero-Backward condition holds for the isolated particle (*λ* = 1685 nm) are shown in Fig. S5. The external and internal radii of the particles are *R*_ext_ = 230 nm and *R*_core_ = 70 nm respectively, and the core has been displaced *x* = 30 nm from the particle center. The separation between particles is 137 nm. The particles, which are not aligned with the propagation direction, ***k*,** make an angle with respect to ***k*** of 20º.

**Supplementary Figure S5 | Scattered intensity, *I*(*θ*_sca_), by eccentric core-shell aggregates**. Scattering diagrams for the polarizations of the incident radiation **(a)** parallel (p-polarization) and **(b)** perpendicular (s-polarization) to the scattering plane for different configurations of eccentric core-shell nanoparticles (see Fig. S6) at the wavelength where the Zero-Backward condition holds for the isolated particle. The inner and outer radii are 70 and 230 nm respectively. The core has been displaced 30 nm along the *x*-axis.

Figure S5 shows how as the number of particle increases so does the scattered intensity. All selected geometries are in the *z*-*x*-plane with core displacement in *x*-direction. The spatial configuration of the particles (see Fig. S6, where a scheme of the different studied configurations can be observed) influences the value of this magnitude, as well as the polarization of the incident radiation. It is a general trend that the scattered intensity for the case of incident radiation linearly polarized parallel to the scattering plane (p-polarization) takes larger values than for the perpendicular one (s-polarization), and viceversa in the case of *z-y* plane geometries. It is clear that the structure that presents better directionality properties corresponds to An6Y, which has been analyzed in detail in the manuscript, see Fig. 7. For this geometry, the scattered intensity takes the highest values and the narrowest pattern is observed, which means better directionality properties.

In order to evidence the importance of the spatial distribution of the core-shell nanoparticles that constitute the aggregates, in Fig. S7, we compare the near- and far-field behavior of the three different analyzed V-shaped chains of 6 nanoparticles (corresponding to geometries An6Y, An6YY and An6YYY in Fig. S6) at the wavelength where the Zero-Backward condition holds for the isolated particle (*λ* = 1685 nm). Figs. S7(a-c) show the near field maps. As an inset, it is represented the spatial configurations of the particles in each case. Figures S7(d-f) plot, for each configuration, the angular distribution of the scattered intensity in the far-field regime for both p- and s-polarizations. Insets show far-field observations of the total scattered intensity (within a solid angle of 30º) for p-polarized incident radiation. From the far-field plots, it is clear that the best directionality properties are obtained for the An6Y configuration. However, the three considered structures are able to guide the incident electromagnetic radiation, as it can be observed by means of the near-field maps.

**Supplementary Figure S6 | Core-shell configurations of a V-shaped chain of 6 core-shell nanoparticles**. Schemes of the different studied configurations of a V-shaped chain of 6 eccentric core-shell nanoparticles. The red and blue arrows represent the directions of propagation and polarization of the incident radiation respectively.

**
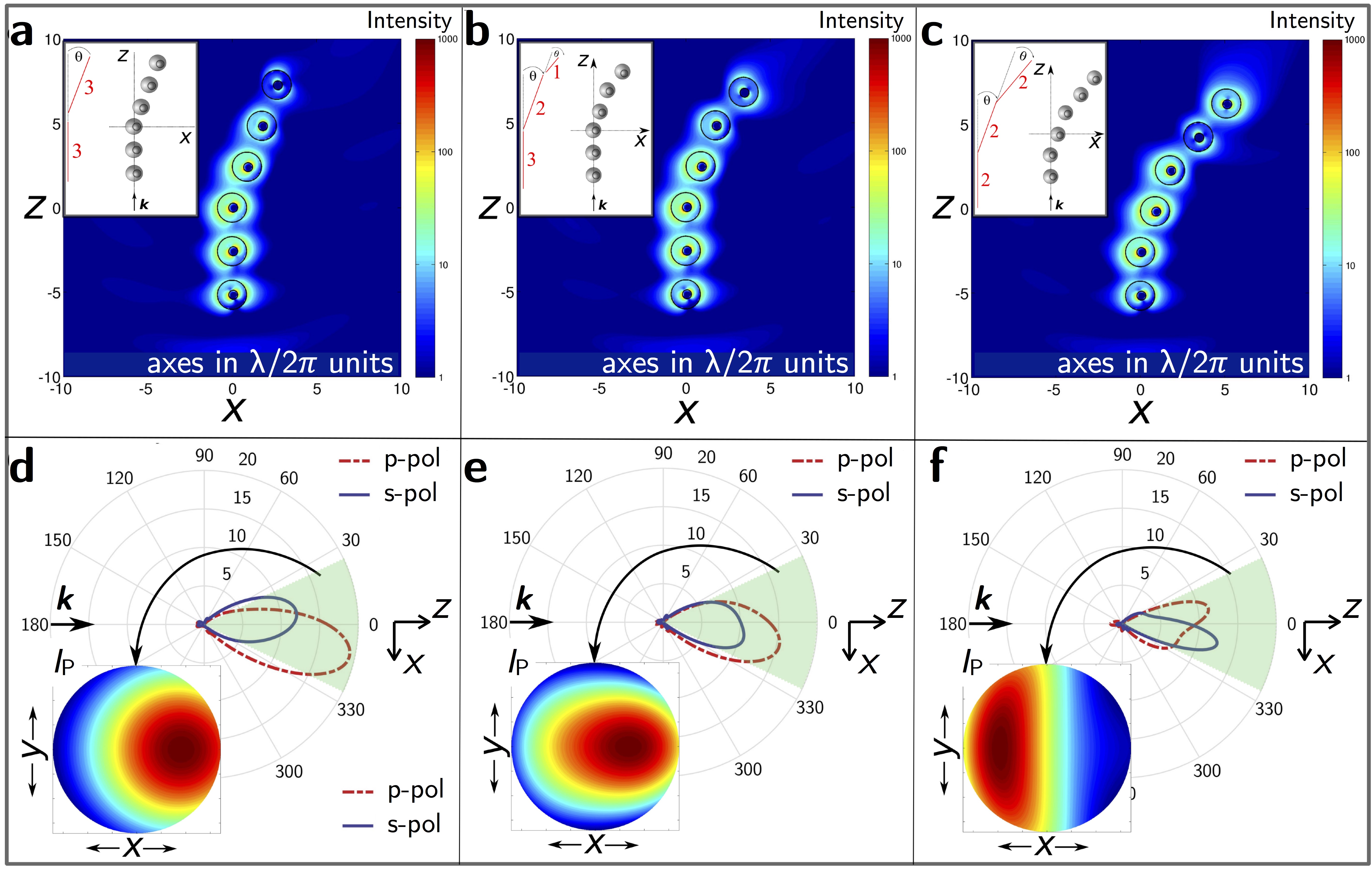
**

**Supplementary Figure S7 | Near- and far-field behavior of a V-shaped chain of 6 core-shell nanoparticles**. **(a to c)** show near field maps of different configurations of a V-shaped chain of 6 core-shell nanoparticles of radii *R*_ext_ = 230 nm and *R*_core_ = 70 nm with the core displaced 30 nm along the *x*-axis (corresponding to geometries An6Y, An6YY and An6YYY in Fig. S6). Insets are schemes of the geometries. The distance between the particles along the *x*-axis is137 nm. The angle between segments of aligned NPs is *θ* = 20º (see insets in (a-c)). The structures have been illuminated with a plane wave propagating along the *z*-axis and polarized along the *x*-axis (p-polarization). The plots correspond to the *z*-*x* plane for the wavelength where the Zero-Backward condition holds for the isolated particle. **(d to f)** Scattering diagrams in the *z*-*x* plane for p-incident polarization (red dashed-dotted line) and s-incident polarization (blue solid line) at the First Kerker’s condition for the geometries shown in the insets of (a-c). Insets show far-field observations of the normalized total scattered intensity, within a solid angle of 30º, for p-polarized incident radiation.

**Supplementary References**

(1) Sanz, J. M. *et al*. Influence of pollutants in the magneto-dielectric response of silicon nanoparticles. *Opt. Lett.* **39,** 3142–3144 (2014).
